# Highly Efficient and Exceptionally Durable Photooxidation Properties on Co_3_O_4_/g-C_3_N_4_ Surfaces

**DOI:** 10.3390/ma16103879

**Published:** 2023-05-22

**Authors:** Yelin Dai, Ziyi Feng, Kang Zhong, Jianfeng Tian, Guanyu Wu, Qing Liu, Zhaolong Wang, Yingjie Hua, Jinyuan Liu, Hui Xu, Xingwang Zhu

**Affiliations:** 1 School of the Environment and Safety Engineering, Institute for Energy Research, Jiangsu University, Zhenjiang 212013, China; 2 College of Environmental Science and Engineering, Yangzhou University, Yangzhou 225009, China; 3 The Key Laboratory of Electrochemical Energy Storage and Energy Conversion of Hainan Province, School of Chemistry and Chemical Engineering, Hainan Normal University, Haikou 571158, China

**Keywords:** photocatalyst, type-II heterojunction, carrier separation, photodegradation

## Abstract

Water pollution is a significant social issue that endangers human health. The technology for the photocatalytic degradation of organic pollutants in water can directly utilize solar energy and has a promising future. A novel Co_3_O_4_/g-C_3_N_4_ type-II heterojunction material was prepared by hydrothermal and calcination strategies and used for the economical photocatalytic degradation of rhodamine B (RhB) in water. Benefitting the development of type-II heterojunction structure, the separation and transfer of photogenerated electrons and holes in 5% Co_3_O_4_/g-C_3_N_4_ photocatalyst was accelerated, leading to a degradation rate 5.8 times higher than that of pure g-C_3_N_4_. The radical capturing experiments and ESR spectra indicated that the main active species are •O_2_^−^ and h^+^. This work will provide possible routes for exploring catalysts with potential for photocatalytic applications.

## 1. Introduction

It is well known that the situation regarding water resources is linked to environmental, social, and economic risks [1,2]. However, large volumes of wastewater dyes and pharmaceutical effluents, including methylene blue, rhodamine B, tetracycline, ciprofloxacin, and so on, have been detected in our daily water bodies [3,4]. As a result, the environmental crisis over water has become one of the top risks facing the world today. Since these pollutants have become a serious threat to humans and ecosystems, there is an urgent need to clean up these colored organic dye pollutants [5]. In order to more effectively mitigate the ecological risks brought by water environment problems, environment-friendly technical methods such as adsorption, electrochemical, and photochemical methods have been proposed. The implementation of these technologies could effectively achieve the effect of purifying wastewater. Among the many technologies, photocatalysis, as a harmless technology for substance conversion, plays an important role in the field of toxic substances conversion. However, as the core of photocatalysis technology, semiconductor photocatalysts are usually limited to green, stable materials that meet the needs of industrial use [6]. To date, several types of semiconductors, such as oxides (TiO_2_ [7], ZnO [8]), nitrides (Ta_3_N_5_ [9], C_3_N_4_ [10,11,12,13,14]), and sulfides (MoS_2_ [15,16], CdS [17,18]) have been developed. In general, as a representative semiconductor material in p-type semiconductors, Co_3_O_4_ is highly sought after by researchers because of its excellent catalytic activity and stability in the field of photocatalysis and its high economic benefits [19,20,21,22]. However, even so, its inherent defects still greatly limit the market expansion and application of such materials, such as their low electron–hole separation rate and relatively limited optical absorption range [23,24]. Based on the above dilemma, the design idea of effectively improving the optical absorption range of Co_3_O_4_ and increasing the separation rate of photogenerated carriers may enable it to meet the demand gap in the field of environmental governance.

For a long time, researchers have also actively carried out a lot of research based on light absorption and carrier separation [25,26]. The implementation of many technical strategies, such as the design of morphologies, the construction of heterostructures, and the modification of precious metals, greatly optimized and improved the photocatalytic performance of Co_3_O_4_. Among them, the construction of semiconductor heterostructures is the most effective way to promote efficient carrier separation and migration and has shown impressive performance in many reports [24,27]. In these heterostructures, the p-type semiconductor Co_3_O_4_ conduction band (CB) and valence band (VB) bend towards vacuum level while the n-type semiconductor bend against vacuum level due to the formation of the built-in electric field in the catalyst and the balance of Fermi energy levels [28,29]. Moreover, the bending is only large at the region far from the depletion region. Driven by the force of the electric field, the charge is further separated efficiently, thus improving the photocatalytic efficiency. At present, the various reported n-type semiconductors that have been used to construct the p-type semiconductor Co_3_O_4_ include g-C_3_N_4_ [11,30], In_2_O_3_ [31], Bi_2_O_3_ [32], and MnO_2_ [33]. Graphitic carbon nitride, a stable polymer semiconductor with a special 2D framework structure of heptazine rings connected via tertiary amines, could form a self-built internal electrostatic field, and the electric field and van der Waals interactions cause photogenerated separation and transport of carriers [29,34]. Moreover, due to its wide band gap, g-C_3_N_4_ exhibits efficient sunlight collection properties. And thanks to its sparse and porous structure, it is also able to easily adsorb and re-degrade pollutants [35]. Therefore, the modification of g-C_3_N_4_-based materials gives us a more practical pathway to enhance the activity of metal oxides. Based on this, we are eager to learn whether the coupling between g-C_3_N_4_ and Co_3_O_4_ could efficiently solve problems in the field of environmental treatment.

In our research, Co_3_O_4_ nanosheets and g-C_3_N_4_ were prepared by a rapid hydrothermal method and a calcination method, respectively, and then Co_3_O_4_/g-C_3_N_4_ nanomaterials were prepared by composing the two. The microstructure and physical and chemical properties of Co_3_O_4_/g-C_3_N_4_ were characterized by several methods, such as HRTEM, XPS, BET, and ESR, and the performance of different mass ratios of Co_3_O_4_/g-C_3_N_4_ on the catalyst photodegradation activity of RhB was investigated under simulated sunlight. The results showed that the photocatalytic activity of Co_3_O_4_/g-C_3_N_4_ was significantly enhanced compared with that of the pure sample, which may be due to the role of the heterojunction established between Co_3_O_4_ and g-C_3_N_4_, which could promote the separation of photogenerated charges and interfacial effects. Finally, a possible charge transfer pathway is proposed based on the experimental results. Our work offers new insights into the application of crystalline semiconductors for the removal of aqueous organic pollutants.

## 2. Materials and Methods

### 2.1. Materials

CO(NH_2_)_2_, Co(NO_3_)_2_·6H_2_O, C_2_H_6_O, NaOH, C_2_H_3_N, C_6_H_15_NO_3_, and C_28_H_31_ClN_2_O_3_ were procured from Sinopharm Chemical Reagent Co., Ltd. (Shanghai, China). (C_6_H_9_NO)_n_ (M.W. ≈ 55,000) was acquired from Aladdin Reagent Co., Ltd. (Shanghai, China). Deionized water was used throughout the experiment. All chemicals were analytically pure and required no further processing.

### 2.2. Synthesis of g-C_3_N_4_

Urea (20 g sample) was added to a 50 mL crucible container and transferred to a muffle furnace and calcined under an air atmosphere. The conditions were set to increase from ambient temperature to 823 K at a rate of 5 K/min for 4 h. After the sample cooled down, the sample was made into powder with a mortar and raised from the initial temperature to 773 K at a rate of 5 K/min for 2 h. The light-yellow powder obtained was g-C_3_N_4_, named CN.

### 2.3. Synthesis of β-Co(OH)_2_

Co(NO_3_)_2_·6H_2_O and (C_6_H_9_NO)_n_ (PVP, M.W. ≈ 55,000) were thoroughly mixed in absolute ethanol and deionized water for 1 h. The mixture was then transferred to a 25 mL Teflon-lined autoclave. It was reacted for 12 h at 473 K before being cooled to room temperature. The pink product was washed several times with deionized water and anhydrous ethanol until the pH of the filtrate reached neutral, and then vacuum dried for 14 h.

### 2.4. Synthesis of Co_3_O_4_

The β-Co(OH)_2_ precursor was heated in a tube furnace at a rate of 5 K/min and kept at 673 K for 2 h. The obtained product was the labeled Co_3_O_4_ nanosheet.

### 2.5. Synthesis of Co_3_O_4_/g-C_3_N_4_

The deionized water was added into the above-prepared Co_3_O_4_ and g-C3N_4_ and mixed with stirring, and a series of Co_3_O_4_/g-C_3_N_4_ mixture samples with different ratios were synthesized by adjusting the mass ratio between Co_3_O_4_ and g-C_3_N_4_. After being rapidly frozen with liquid nitrogen, the samples were dried in a freeze-drying oven for 72 h.

## 3. Results and Discussion

### 3.1. Microscopic Morphology and Chemical Structure Characterization

**Figure 1:**
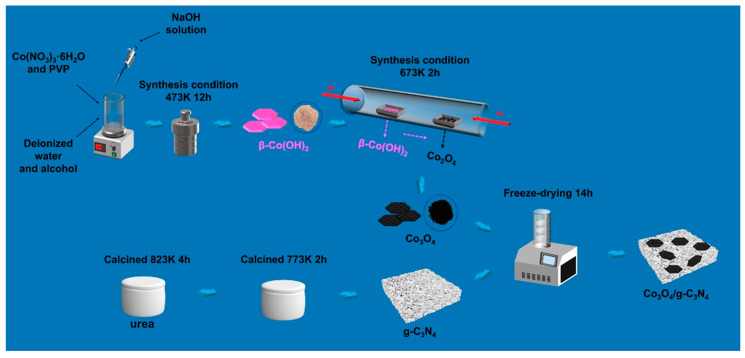
Schematic graph of the synthesis route of Co_3_O_4_/g-C_3_N_4_.

The synthesis route of the Co_3_O_4_/g-C_3_N_4_ sample is displayed in Figure 1. Here, urea was thermally oxidized to obtain g-C_3_N_4_ sample. At the same time, β-Co(OH)_2_ was prepared by solvothermal reaction as a precursor of Co_3_O_4_. Eventually, the Co_3_O_4_/g-C_3_N_4_ heterojunction was produced by liquid nitrogen-assisted thermal oxidation.

**Figure 2:**
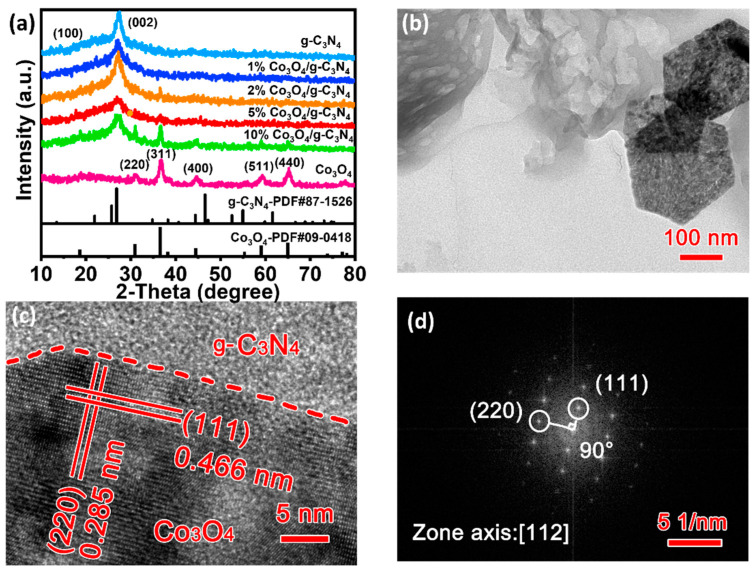
(**a**) XRD patterns; (**b**) TEM; (**c**,**d**) HRTEM and corresponding FFT images of 5% Co_3_O_4_/g-C_3_N_4_.

X-ray diffraction (XRD) was used to analyze crystallographic structures of samples in Figure 2a. It can be seen that g-C_3_N_4_ has broad peaks at 13.2° and 27.6°, corresponding to the (100) and (002) crystal planes of g-C_3_N_4_ (JCPDS No. 87-1526), and the Co_3_O_4_/g-C_3_N_4_ catalyst exhibits only very weak Co_3_O_4_ diffraction peaks due to the low loading percentage of the Co_3_O_4_ catalyst (JCPDS No. 09-0418). Five characteristic peaks were identified at 2θ = 31.3° (d = 2.86 Å), 36.85° (d = 2.44 Å), 55.64° (d = 1.65 Å), 59.35° (d = 1.56 Å), and 65.22° (d = 1.43 Å) corresponding to (220), (311), (422), (511), and (440) as cubic Co_3_O_4_ crystal faces [11]. The transmission electron microscopy (TEM) image of 5% Co_3_O_4_/g-C_3_N_4_ was shown in Figure 2b, where Co_3_O_4_ nanosheets of about 150–200 nm in size can be observed on the surface of g-C_3_N_4_. HRTEM and corresponding FFT studies were performed for 5% Co_3_O_4_/g-C_3_N_4_ (Figure 2c,d), and the lattice stripes with spacing of 0.285 and 0.466 nm correspond to the (220) and (111) crystal planes of Co_3_O_4_ (JCPDS No. 09-0418) [20]. In summary, a clear interface existed between Co_3_O_4_ and g-C_3_N_4_, and the interfacial contact facilitates the rapid transfer of photogenerated charges.

**Figure 3:**
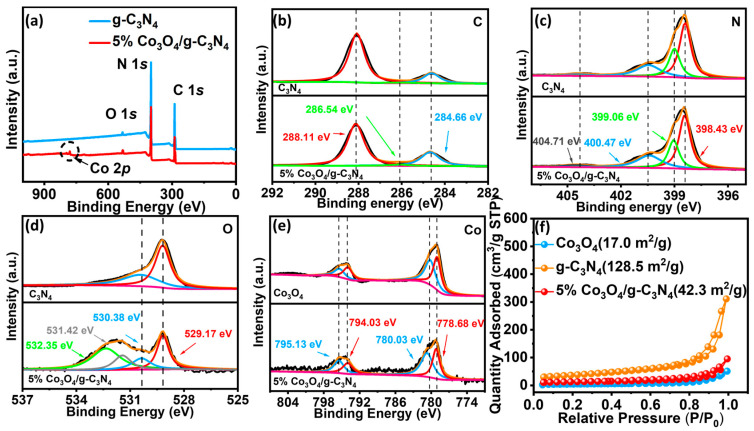
XPS spectra of (**a**) samples; (**b**) C 1s; (**c**) N 1s; (**d**) O 1s; (**e**) Co 2p; (**f**) N_2_ adsorption–desorption technique.

The analysis of X-ray photoelectron spectroscopy (XPS) provides insight into the surface composition and chemical changes in each sample. From the full survey spectra of samples in Figure 3a, it was found that Co, C, N, and O elements were detected in 5% Co_3_O_4_/g-C_3_N_4_, and the molar ratio of C:N:O:Co in 5% Co_3_O_4_/g-C_3_N_4_ was about 48:49.6:2:0.3, which further confirmed the complexation of Co_3_O_4_ with g-C_3_N_4_. The high-resolution XPS spectra of the C 1s spectra at energies of 288.11 eV, 286.54 eV, and 284.66 eV belong to the C-O bond and the N-C=N bond in Figure 3b and Figure S1. The peaks of the N 1s spectra (Figure 3c) are located at 404.71 eV, 400.47 eV, 399.06 eV, and 398.43 eV, respectively, which can be attributed to sp2-hybridized nitrogen C-N=C, tertiary nitrogen N-(C)_3_, and primary nitrogen H-N-(C)_2_ [10,12]. The peaks of the O 1s spectra can be shown in Figure 3d, except peaks at 530.38 and 529.17 eV and at 532.35 and 531.42 eV can be found, which originate from the O-C=O and C-O groups produced at the interface of Co_3_O_4_ and g-C_3_N_4_ [33,36]. The Co 1s energy spectra of Co_3_O_4_ and the Co 2p energy spectra of 5% Co_3_O_4_/g-C_3_N_4_ samples (Figure 3e) showed four characteristic peaks at 795.13 eV, 794.03 eV, 780.03 eV, and 778.68 eV, which can be ascribed to the Co-O and Co=O bonds [27,37]. The effect of photocatalytic degradation is influenced by the specific surface area of the material, and the surface area of different samples was investigated by the N_2_ adsorption–desorption technique (BET). The g-C_3_N_4_ exhibits a typical type IV isotherm with H3-type hysteresis loops, and its mesoporous structure may be due to the stacking of the g-C_3_N_4_ (Figure 3f). The surface area of g-C_3_N_4_ is about 128.5 m^2^/g as calculated by the model that comes with the instrument. The higher specific surface area is attributed to the large-scale nanosheet morphology of g-C_3_N_4_. The specific surface area of the 5% Co_3_O_4_/g-C_3_N_4_ composite was slightly decreased after combining with Co_3_O_4_, probably since the decrease in specific surface area caused by the interfatial contact between Co_3_O_4_ and g-C_3_N_4_. The interfacial effect of 5% Co_3_O_4_/g-C_3_N_4_ promotes the adsorption of pollutants by the catalyst, and the abundant active sites are conducive to efficient photocatalytic reactions.

### 3.2. Performance Analysis and Kinetics Study of RhB Degradation by Photocatalytic Application

**Figure 4:**
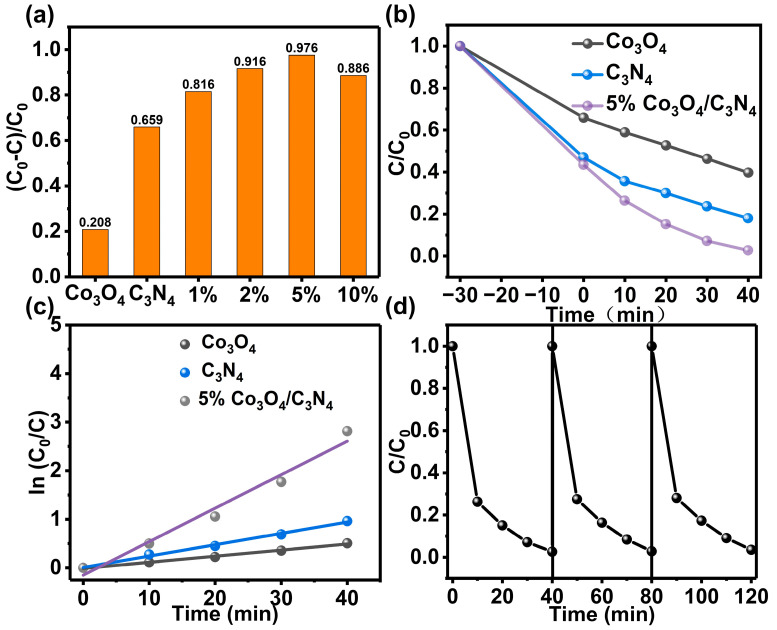
(**a**) Photodegradation rate of RhB by different samples under simulated sunlight for 40 min; (**b**) g-C_3_N_4_, Co_3_O_4_ and 5% Co_3_O_4_/g-C_3_N_4_ photocatalytic degradation of RhB with time; (**c**) photocatalytic reaction kinetics; (**d**) stability test of 5% Co_3_O_4_/g-C_3_N_4_.

The photodegradation RhB activity of different proportions of samples is usually tested under simulated sunlight conditions. As shown in Figure 4a, the degradation effect of RhB after 40 min under different sample light conditions, demonstrates that the heterogeneous combination of g-C_3_N_4_ and Co_3_O_4_ effectively promoted the photocatalytic reaction. Among them, the degradation of RhB by 5% Co_3_O_4_/g-C_3_N_4_ can reach 97.6%. At low concentrations, more Co_3_O_4_ facilitates the rapid carrier transfer and promotes the photocatalytic degradation reaction. However, when the concentration is high, Co_3_O_4_ covers the surface of g-C_3_N_4_, which hinders its light absorption and obscures the active site, thus causing a decrease in the reaction activity. Figure 4b shows the variation of different proportions in the samples, photocatalytic degradation of RhB over time, which more clearly confirms that the catalytic ability of 5% Co_3_O_4_/g-C_3_N_4_ is stronger than additional two monomeric catalysts. Based on the above characterization, a reaction kinetic model was established (Figure 4c), and the perfect linear relationship between In(C_0_/C) and irradiation time indicates that the photocatalytic reaction is the quasi-primary reaction; g-C_3_N_4_, Co_3_O_4_ and 5% Co_3_O_4_/g-C_3_N_4_ have rate constants k values of 0.024 min^−1^, 0.0126 min^−1^, and 0.0703 min^−1^, respectively. The degradation efficiency of 5% Co_3_O_4_/g-C_3_N_4_ is approximately 3 times that of g-C_3_N_4_ and 5.58 times that of Co_3_O_4_. The Co_3_O_4_/g-C_3_N_4_ exhibited better photocatalytic activity than most of the reported photoreduction systems under similar conditions (Table S1). In addition, the repeatability of the 5% Co_3_O_4_/g-C_3_N_4_ material was tested in Figure 4d. It can be shown that the performance of 5% Co_3_O_4_/g-C_3_N_4_ did not show significant degradation after three cycles, which proved the excellent stability of the composite through interfacial compounding.

**Figure 5:**
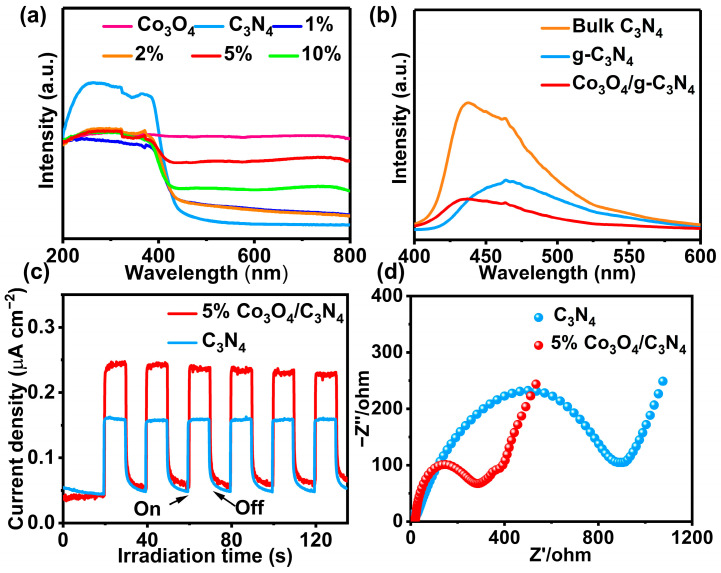
(**a**) DRS spectra, (**b**) PL spectra; (**c**) photocurrent responses, and (**d**) EIS measurement of samples.

Testing the UV–vis diffuse reflectance spectroscopy (DRS) of catalysts can provide insight into their light absorption capabilities and help in studying their optical properties. It can be seen from Figure 5a, the absorption edge of g-C_3_N_4_ is about 450 nm, and there is almost no response in visible region beyond 450 nm. However, the absorption of 5% Co_3_O_4_/g-C_3_N_4_ is significantly stronger in visible light due to the interfacial effect formed between Co_3_O_4_ and g-C_3_N_4_, which helps to improve the photocatalytic activity of the catalyst [38,39,40]. The photoluminescence (PL) spectra show that the fluorescence intensities of g-C_3_N_4_ and Co_3_O_4_ were significantly higher than 5% Co_3_O_4_/g-C_3_N_4_, which indicates a higher complexation rate of photogenerated carriers on Co_3_O_4_ and g-C_3_N_4_ [41,42] (Figure 5b). To further demonstrate that 5% Co_3_O_4_/g-C_3_N_4_ has better photogenerated charge separation efficiency, the time-dependent photocurrent of samples was analyzed. The 5% Co_3_O_4_/g-C_3_N_4_ catalyst exhibited a higher photocurrent response intensity compared with single g-C_3_N_4_, which indicates that the composite catalyst promotes the separation and transfer of photogenerated electron–hole pairs in Figure 5c. Furthermore, the 5% Co_3_O_4_/g-C_3_N_4_ catalyst also has a smaller arc radius in the Nyquist plot of electrochemical impedance spectroscopy (EIS), which further suggests that the 5% Co_3_O_4_/g-C_3_N_4_ catalyst has better photogenerated carrier separation efficiency (Figure 5d and Figure S2) [43,44]. Therefore, the stronger photocurrent response and the smaller charge transfer impedance suggest that the photogenerated electron–hole pairs can be effectively separated in 5% Co_3_O_4_/g-C_3_N_4_.

**Figure 6:**
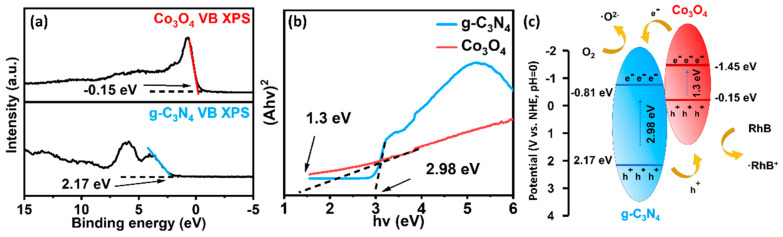
(**a**) XPS-VB spectra of Co_3_O_4_ and g-C_3_N_4_; (**b**) Tauc plots of Co_3_O_4_ and g-C_3_N_4_; (**c**) Co_3_O_4_ and g-C_3_N_4_ electronic band structures and schematic diagram of electrons transfer paths.

Based on the XPS valence band (XPS-VB) spectral analysis, the VB maxima of Co_3_O_4_ and g-C_3_N_4_ can be determined to be −0.15 and 2.17 eV, respectively (Figure 6a); therefore, the conduction band (CB) minima of Co_3_O_4_ and g-C_3_N_4_ can be easily calculated as −2.92 and −3.08 eV. By analyzing the DRS spectra, the bandgaps (Eg) of Co_3_O_4_ and g-C_3_N_4_ were obtained to be 1.3 and 2.98 eV, respectively (Figure 6b). Based on the above analysis, a type-II heterojunction [2] photocatalytic mechanism is proposed in Figure 6c. The 5% Co_3_O_4_/g-C_3_N_4_ photocatalyst was excited beneath light irradiation and generates electron and hole pairs, and transferred the electrons from the CB of Co_3_O_4_ to g-C_3_N_4_. Thanks to the intrinsic force field shaped by interface contact between Co_3_O_4_ and g-C_3_N_4_, whereas the holes on the VB of g-C_3_N_4_ are often transferred to Co_3_O_4_. The Co_3_O_4_ can produce more photogenerated electrons on the CB of g-C_3_N_4_ that can promote the conversion of superoxide radicals (•O_2_^−^) and accelerate the conversion of RhB to the subsequent mineralization products.

**Figure 7:**
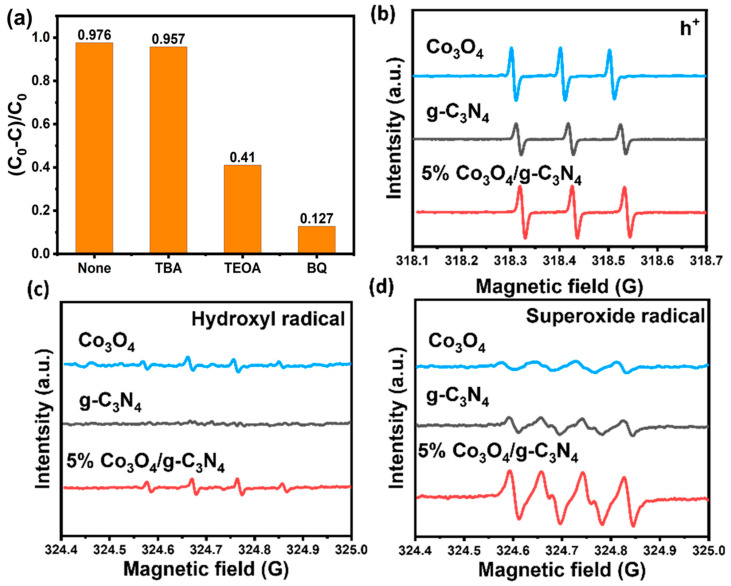
(**a**) Photodegradation rate of RhB by 5% Co_3_O_4_/g-C_3_N_4_ under different scavengers within 40 min; ESR spectra of g-C_3_N_4_, Co_3_O_4_ and 5% Co_3_O_4_/g-C_3_N_4_ (**b**) holes; (**c**) hydroxyl radicals; (**d**) superoxide radicals.

To explore the active species in the 5% Co_3_O_4_/g-C_3_N_4_ photocatalytic degradation of RhB, a series of free radical capturing experiments was performed (Figure 7a). Tertiary butanol (TBA), triethanolamine (TEOA), and benzoquinone (BQ) were used as the capture agents of •OH, h^+^ and •O_2_^−^. After 40 min of light irradiation, it was found that the degradation efficiency of RhB by 5% Co_3_O_4_/g-C_3_N_4_ was significantly inhibited by the addition of TEOA and BQ, while the degradation effect did not change significantly after the addition of TBA. The radical capturing experiments indicated that the active species within the degradation of RhB by 5% Co_3_O_4_/g-C_3_N_4_ were in the main •O_2_^−^ and h^+^, however not •OH. In order to further verify the results, an electron spin resonance (ESR) analysis was carried out. After 10 min of irradiation with a Xe lamp (300 W), the 5% Co_3_O_4_/g-C_3_N_4_ surface produced strong •O_2_^−^ and h^+^ signals (Figure 7b,c), while almost no signal of •OH appeared (Figure 7d), proving that •O_2_^−^ and h^+^ are the main reactive groups in the reaction system, which is in line with the results of the radical capturing experiments. In addition, it was often found that the signals of •O_2_^−^ and h^+^ on the surface of 5% Co_3_O_4_/g-C_3_N_4_ significantly exceeded those of g-C_3_N_4_, indicating that the created heterojunction will higher separate the photogenerated carriers, confirming the results of the previous analysis.

## 4. Conclusions

In summary, a novel type-Ⅱ heterojunction photocatalyst (Co_3_O_4_/g-C_3_N_4_) was prepared by simple hydrothermal and calcination methods and used to efficiently degrade RhB in water. The experimental results showed that the Co_3_O_4_/g-C_3_N_4_ photocatalyst had robust photocatalytic degradation activity toward RhB under light irradiation. The DRS, PL, time-dependent photocurrent, and EIS analyses revealed that the type-II heterojunction structure effectively reduced the composite rate of photogenerated electrons and holes, and therefore the holes in VB of Co_3_O_4_ and therefore the electrons in CB of g-C_3_N_4_ were utilized to reinforce the oxidation–reduction ability of the photocatalyst, which resulted in the speedy degradation of pollutants. Among them, the best degradation potency was achieved by a 5% Co_3_O_4_/g-C_3_N_4_ photocatalyst, and the RhB degradation potency was increased by 48% compared with the g-C_3_N_4_ photocatalyst. This study provides some reference data for the development of different heterojunction photocatalysts for the degradation of organic pollutants.

## Data Availability

Raw data are available upon request.

## Supplementary Materials

The following supporting information can be downloaded at: https://www.mdpi.com/article/10.3390/ma16103879/s1. Characterization of the photocatalysts. Photoelectrochemical test. Photocatalytic degradation activity test. Figure S1. The high-resolution O 1s spectra of Co_3_O_4_. Figure S2. EIS spectra under light conditions. Table S1. Common photocatalyst and the effect of degrading organic pollutants in water. Refs [45,46,47,48,49,50,51,52,53,54] were cited in the Supplementary Materials.
