# Supplementary material for: Highly Efficient and Exceptionally Durable Photooxidation Properties on Co3O4/g-C3N4 Surfaces
Source: Materials (Basel). 2023 May 22;16(10):3879. doi: 10.3390/ma16103879 (PMC10223640; doi:10.3390/ma16103879)
Supplement: Supplementary file 1 [file materials-16-03879-s001.zip › materials-2330861-supplementary.pdf]

Supplementary Information

Highly Efficient and Exceptionally Durable Photooxidation Properties on Co_3O_4/g-C_3N_4 Surfaces

Yelin Dai[1], Ziyi Feng[1], Kang Zhong[1], Jianfeng Tian[1], Guanyu Wu[1], Qing Liu[2], Zhaolong Wang[1], Yingjie Hua[3], Jinyuan Liu[1], Hui Xu[1] and Xingwang Zhu[2,]*

1 School of the Environment and Safety Engineering, Institute for Energy Research, Jiangsu University, Zhenjiang 212013, PR China

2 College of Environmental Science and Engineering, College of Mechanical Engineering, Yangzhou University, Yangzhou 225009, PR China

3 The Key Laboratory of Electrochemical Energy Storage and Energy Conversion of Hainan Province, School of Chemistry and Chemical Engineering, Hainan Normal University, Haikou 571158, PR China

*Corresponding author.

E-mail addresses: zxw@yzu.edu.cn (X. Zhu)

Characterization of the photocatalysts

X-ray diffraction (XRD, D8 X, Bruker) patterns were recorded with Cu Kα radiation (λ = 1.54178 Å). X-ray photoelectron spectroscopy (ESCALAB MKII X, Thermo Fisher) measurements were performed. The field emission scanning electron microscopy (SEM) images were performed by using a JEOL-JSM-7800F. Transmission electron microscopy (TEM) and high-resolution TEM images were obtained by using a JEOL-2010. UV-vis diffuse reflectance spectroscopy (DRS) was recorded on a UV-3600 plus (Shimadzu, Japan). Fourier transform infrared (FT-IR) spectra were acquired on a Nicolet iS-50 spectrometer with KBr tablets, scanning from 4000 to 400 cm^{-1} at room temperature (Thermo Fisher Scientific, America). Room temperature photoluminescence (PL) were recorded on a QM4m fluorescence spectrophotometer (QM4m, PTI). Electron spin resonance (ESR) patterns were recorded on a Bruker model ESR JES-FA200 spectrometer in methanol and water, respectively.

Photoelectrochemical test

An electrochemical workstation (Chenhua Instruments Company, China, 660B) was used to perform electrochemical measurements using a three-electrode system. In the three-electrode system, the reference electrode was a silver and silver chloride, and the platinum wire was used as the counter electrode. The working electrode was prepared as follows: 5 mg of photocatalyst powder was dispersed in 0.5 mL ethylene glycol, then 20 μL of the dispersed solution was coated in a 10 × 5 mm indium tin oxide (ITO) glass and dried under 60°C to remove ethanol for 12 hours. The electrolyte solution is 0.2 M Na_2SO_4. The light source is a 500 W Xenon lamp (PLS-FX300HU, Beijing Perfectlight). Electrochemical impedance spectra (EIS) measurements were performed in 0.1 mol/L KCl solution containing 5 mM $Fe(CN)_6^{3-}/Fe(CN)_6^{4-}$.

Photocatalytic degradation activity test

Photocatalytic degradation of RhB: 100 mL of 10^{-5} M RhB solution containing 10 mg of catalyst was poured into a 100 mL reactor and kept in magnetic stirring throughout the experiment. The solution was first allowed to react darkly for 30 min to reach adsorption-desorption equilibrium, and then irradiated with a 300 W xenon lamp (PLS-SXE300, Porphyry) for degradation experiments. Every 10 min, 3 mL of supernatant was removed from the solid catalyst by high-speed centrifugation (8000 r/min), and the absorbance of the supernatant at 554 nm was measured by UV spectrophotometer.

Photocatalytic cycle: The photocatalytic cycle is similar to the photocatalytic degradation of RhB. After each cycle, the photocatalyst is washed three times with

deionized water and anhydrous ethanol and dried under vacuum, and then a new cycle of reaction testing is started.

To investigate the active species produced in the photodegradation process of photocatalytic, hydroxyl radicals (•OH), holes (h^+) and superoxide radical (•O^{2-}) were probed by adding 5 mM Tertiary butanol (TBA), triethanolamine (TEOA) and benzoquinone (BQ), respectively.

Figure and Table

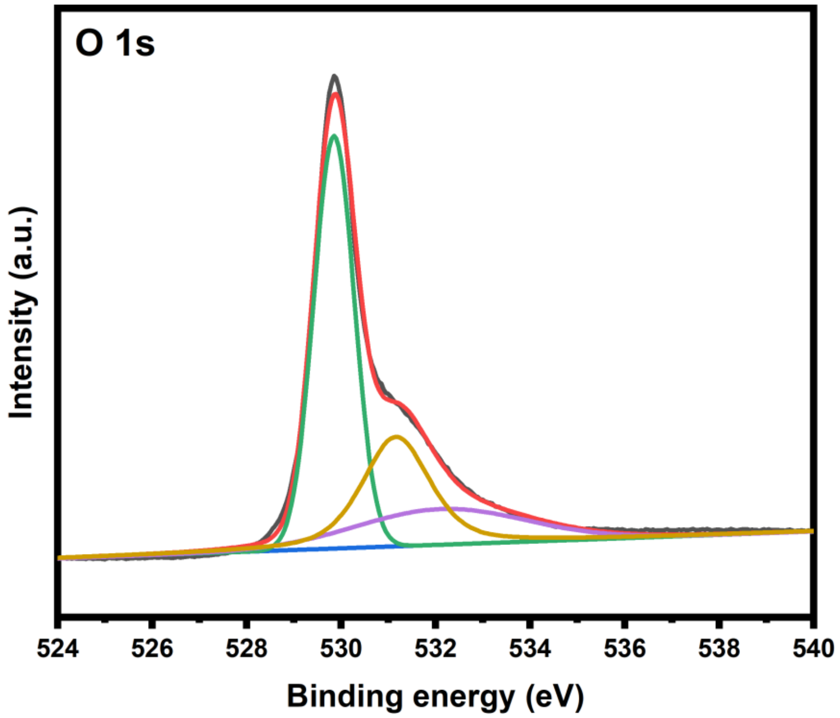

Figure S1. The high-resolution O 1s spectra of Co_3O_4.

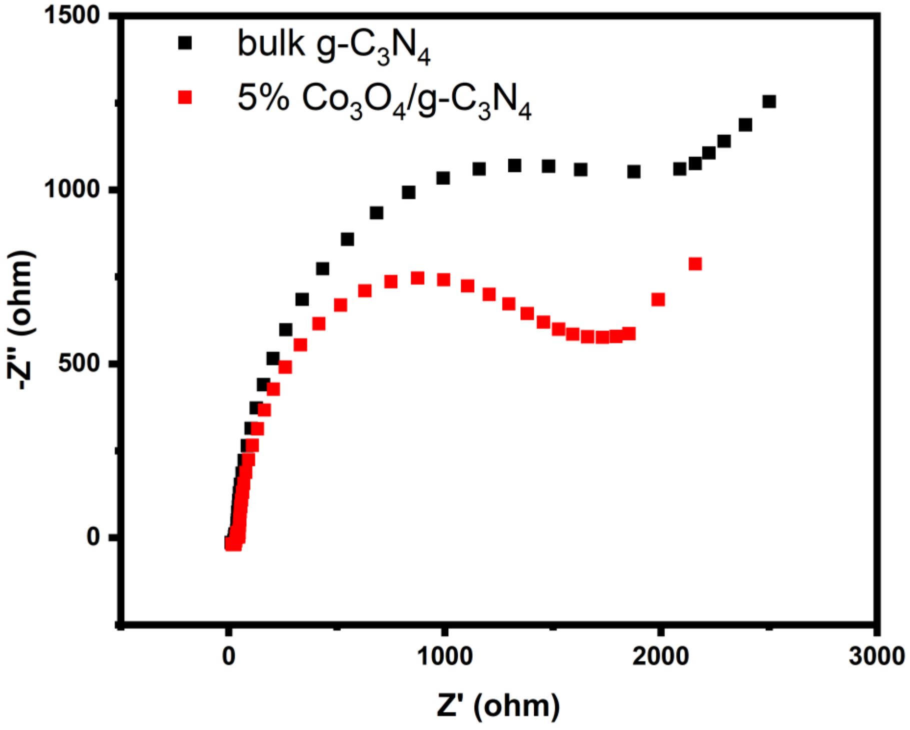

Figure S2. EIS spectra under light conditions.

Table S1. Common photocatalyst and the effect of degrading organic pollutants in water.

Photocatalyst	Reaction medium	Degradation rate (min^{-1})	Ref.
Co_3O_4/g-C_3N_4	300 W Xe-lamp, 10 mg catalyst, 100 mL 10^{-5} M RhB	0.0703	This work
P-Doped g-C_3N_4	300 W Xe-lamp, 50 mg catalyst, 100 mL 10mg/L RhB	0.0659	S1
g-C_3N_4	300 W Xe-lamp, 20 mg catalyst, 100 mL 10mg/L RhB	0.051	S2
CQDs/Sb_2WO_6	300 W Xe-lamp, 100 mg catalyst, 100 mL 10mg/L RhB	0.01346	S3
MIL-125(Ti)/BiOI	250 W Xe-lamp, 15 mg catalyst, 100 mL 20mg/L RhB	0.034	S4
MoS_2	300 W Xe-lamp, 50 mg catalyst, 50 mL 10mg/L RhB	0.212	S5
$BiFeO_3$@B-rGO	300 W Xe-lamp, 20 mg catalyst, 40 mL 10mg/L RhB	0.0241	S6
$CoFe_2O_4$@Bi_2WO_6 @BiOBr	400 W metal halide lamp, 65 mL 10mg/L RhB	0.01208	S7
BiOCl	86 W 420 nm LED, 50 mg catalyst, 100 mL 10mg/L RhB	0.1867	S8
$SbVO_4$/g-C_3N_4	300 W Xe-lamp, 50 mg catalyst, 100 mL 10mg/L RhB	0.0587	S9
O-g-C_3N_4/SnO_2	300 W Xe-lamp, 100 mg catalyst, 100 mL 10mg/L RhB	0.0458	S10

References

[S1] J. Feng, D. Zhang, H. Zhou, M. Pi, X. Wang, S. Chen, Coupling P Nanostructures with P-Doped g-C_3N_4 As Efficient Visible Light Photocatalysts for H_2 Evolution and RhB Degradation, ACS Sustainable Chemistry & Engineering, 6 (2018) 6342-6349

[S2] G. Liu, M. Liao, Z. Zhang, H. Wang, D. Chen, Y. Feng, Enhanced photodegradation performance of Rhodamine B with g-C_3N_4 modified by carbon nanotubes, Separation and Purification Technology, 244 (2020) 116618.

[S3] W. Li, Z. Wang, Y. Li, J.B. Ghasemi, J. Li, G. Zhang, Visible-NIR light-responsive 0D/2D CQDs/Sb_2WO_6 nanosheets with enhanced photocatalytic degradation performance of RhB: Unveiling the dual roles of CQDs and mechanism study, J Hazard Mater, 424 (2022) 127595.

[S4] Q. Hu, J. Dong, Y. Chen, J. Yi, J. Xia, S. Yin, H. Li, In-situ construction of bifunctional MIL-125(Ti)/BiOI reactive adsorbent/photocatalyst with enhanced removal efficiency of organic contaminants, Applied Surface Science, 583 (2022) 152423.

[S5] P. Nandigana, S. Mahato, M. Dhandapani, B. Pradhan, B. Subramanian, S.K. Panda, Lyophilized tin-doped MoS_2 as an efficient photocatalyst for overall degradation of Rhodamine B dye, Journal of Alloys and Compounds, 907 (2022) 164470.

[S6] R. Preetha, M. Govinda raj, E. Vijayakumar, M.G. Narendran, E. Varathan, B. Neppolian, U. Jeyapaul, A. John Bosco, Promoting photocatalytic interaction of boron doped reduced graphene oxide supported $BiFeO_3$ nanocomposite for visible-light-induced organic pollutant degradation, Journal of Alloys and Compounds, 904 (2022) 164038.

[S7] Y. Chen, X. Su, M. Ma, Y. Hou, C. Lu, F. Wan, Y. Ma, Z. Xu, Q. Liu, M. Hao, R. Pan, Y. Liu, One-dimensional magnetic flower-like $CoFe_2O_4$@Bi_2WO_6@BiOBr composites for visible-light catalytic degradation of Rhodamine B, Journal of Alloys and Compounds, 929 (2022) 167297.

[S8] H.H. Naing, Y. Li, J.B. Ghasemi, J. Wang, G. Zhang, Enhanced visible-light-driven photocatalysis of in-situ reduced of bismuth on BiOCl nanosheets and montmorillonite loading: Synergistic effect and mechanism insight, Chemosphere, 304 (2022) 135354.

[S9] C. Li, Y. Zhao, J. Fan, X. Hu, E. Liu, Q. Yu, Nanoarchitectonics of S-scheme 0D/2D $SbVO_4$/g-C_3N_4 photocatalyst for enhanced pollution degradation and H_2 generation, Journal of Alloys and Compounds, 919 (2022) 165752.
[S10] Y. Chen, Y. Jiang, B. Chen, H. Tang, L. Li, Y. Ding, H. Duan, D. Wu, Insights into the enhanced photocatalytic activity of O-g-C_3N_4 coupled with SnO_2 composites under visible light irradiation, Journal of Alloys and Compounds, 903 (2022) 163739.
